# Long-term oncologic outcomes after laparoscopic versus open rectal cancer resection: a high-quality population-based analysis in a Southern German district

**DOI:** 10.1007/s00464-018-6148-6

**Published:** 2018-04-02

**Authors:** Teresa Draeger, Vinzenz Völkel, Michael Gerken, Monika Klinkhammer-Schalke, Alois Fürst

**Affiliations:** 1 Universität Regensburg, Universitätsstraße 31, 93053 Regensburg, Germany; 2 Tumorzentrum Regensburg, Institut für Qualitätssicherung und Versorgungsforschung der Universität Regensburg, Am BioPark 9, 93053 Regensburg, Germany; 3 Caritas Krankenhaus St. Josef Regensburg, Klinik für Allgemein-, Viszeral-, Thoraxchirurgie und Adipositasmedizin, Landshuter Str. 65, 93053 Regensburg, Germany

**Keywords:** Bowel cancer, Minimal invasive surgery, Health services research, Registries, Cohort studies

## Abstract

**Background:**

An increasing number of rectal carcinoma resections in Germany and worldwide are performed laparoscopically. The recently published COLOR II trial demonstrated the oncologic safety of this surgical approach. It remains unclear whether these findings can be transferred to clinical practice.

**Patients and methods:**

This population-based retrospective cohort study aimed to evaluate 5-year overall, relative, disease-free, and local recurrence-free survival of rectal cancer patients treated by open surgery and laparoscopy. Data from a southern German region of 1.1 million inhabitants were collected by an official clinical cancer registry. All primary non-metastatic rectal adenocarcinoma cases with surgery between 2004 and 2013 were eligible for inclusion. To compare survival rates, Kaplan–Meier analyses, relative survival models, and multivariate Cox regression were applied; a sensitivity analysis assessed bias by exclusion.

**Results:**

Finally, 1507 patients with a median follow-up time of 7.1 years were included. Of these patients, 28.4% underwent laparoscopic procedures, with an increasing rate over time. Patients with tumors of the upper or middle rectum, younger patients, and patients of specialized colorectal cancer centers were more likely to undergo laparoscopy. After 5 years, 80.4% of laparoscopy patients were still alive, compared to 68.6% in the open group (*p* < 0.001). Moreover, laparoscopy was associated with superior local recurrence-free survival rates. This advantage was also significant in multivariate analysis (HR 0.70, 95% CI 0.52–0.92).

**Conclusion:**

Laparoscopic rectal cancer surgery can be considered safe in daily clinical practice. This should be confirmed by future studies outside the setting of randomized trials.

According to a survey conducted by the World Health Organization (WHO), 1.4 million people worldwide were diagnosed with colon or rectal cancer in 2012 [1]. In Germany, colorectal cancer is the third most common cancer within the male and the second most common tumor within the female population [2]. The only effective way to treat colorectal carcinoma includes radical surgical resection of the tumor. For many years, open surgery was the gold standard for rectal resections, and even today, many surgeons still prefer the conventional approach. Despite different randomized controlled trails (RCTs) [3] proclaiming the oncologic safety of laparoscopic procedures, there is an ongoing discussion on the topic [4]. More population-based surveys examining long-term survival after rectal cancer surgery in real-life situations are required. In Germany, rectal cancer therapy is highly influenced by evidence-based guidelines [5], which ensure a patient’s adequate treatment regardless of their social or economic status. From a scientific point of view, these are ideal conditions to objectively examine oncologic outcomes after tumor resection in daily clinical practice. As one of the biggest national cancer registries, the independent University of Regensburg Institute for Quality Control and Health Services Research [6] collects detailed information on all cancer patients within a cohesive population of 1.1 million people [7] and thus ensures representative results.

## Patients and methods

The aim of this retrospective cohort study was to compare overall, relative, disease-free, and local recurrence-free survival rates after laparoscopic and open rectal carcinoma surgery. According to the National Cancer Institute, disease-free survival was defined as time from surgery to the presence of locoregional recurrence, distant metastases, or death from any cause as the event of interest [8]. All analyses are based on data gathered from an official German cancer registry (Tumor Center Regensburg/University of Regensburg Institute for Quality Control and Health Services Research [6]). Trained documentation officers systematically collect medical records of all patients registered within a large political district comprising 1.1 million inhabitants who have been diagnosed with a malignant tumor. Before they can do so, all patients have to provide their consent in written form. Information on life status is regularly updated by official authorities or death certificates issued by the public health service. For the purpose of this study, all patients with major elective resections (German Procedure Classification, OPS, 5-484, 5-485, and 5-456 [9]) of histologically confirmed primary, non-metastatic rectal adenocarcinoma between January 1, 2004 and December 31, 2013 were eligible for inclusion in the analysis. Details on each patient include demographics, oncologic comorbidities, tumor characteristics, surgical procedure, and pre- and postoperative chemoradiotherapy (Table 1). The latter two variables were not classified in the usual way as “yes” or “no”, but rather adapted to the requirements of the recent German colorectal cancer treatment guideline [5]. This accounts for the fact that, for example, it is perfectly appropriate for a TNM stage I patient not to receive neoadjuvant radiotherapy, while the situation of a stage III patient can be improved considerably by administering such an additional treatment. The variables “preoperative therapy” and “postoperative therapy” as used in this study, featuring values like “no therapy according to guidelines” or “no therapy in contradiction to guidelines” (Table 1), form homogenous groups with regard to the expectable impact on patients’ health. Therefore, these variables can be adjusted for in a multivariate model, as described below, without the need to stratify by indication group.

**Table 1:** Baseline characteristics of the study population according to surgical access

		Open (*n* = 1079)		Laparoscopic (*n* = 428)		Total (*n* = 1507)		*χ* ^2^
		*n*	%	*n*	%	*n*	%	*p* value
Gender	Male	688	63.8	273	63.8	961	63.8	0.994
	Female	391	36.2	155	36.2	546	36.2	
Age (years)	≤ 64	385	35.7	200	46.7	585	38.8	0.001
	65–77	475	44.0	173	40.4	648	43.0	
	≥ 78	219	20.3	55	12.9	274	18.2	
Previous carcinomas	Yes	41	3.8	19	4.4	60	4.0	0.567
	No	1038	96.2	409	95.6	1447	96.0	
Synchronous carcinomas	Yes	27	2.5	7	1.6	34	2.3	0.307
	No	1052	97.5	421	98.4	1473	97.7	
Location	Upper third	399	37.0	179	41.8	578	38.4	0.001
	Middle third	332	30.8	157	36.7	489	32.4	
	Lower third	348	32.3	92	21.5	440	29.2	
Grading	G1/2	922	85.4	379	88.6	1301	86.3	0.114
	G3/4	157	14.6	49	11.4	206	13.7	
TNM stage (UICC)	I	264	24.5	107	25.0	371	24.6	0.256
	II	348	32.3	120	28.0	468	31.1	
	III	467	43.3	201	47.0	668	44.3	
Hospital classification	Center	910	84.3	395	92.3	1305	86.6	0.001
	Other hospitals	169	15.7	33	7.7	202	13.4	
Resection group	Sphincter preservation	780	72.3	372	86.9	1152	76.4	0.001
	No sphincter preservation	248	23.0	50	11.7	298	19.8	
	Extended resection	51	4.7	6	1.4	57	3.8	
Preoperative therapy	No neoadjuvant therapy according to guidelines	504	46.7	200	46.7	704	46.7	0.010
	Neoadjuvant therapy	398	36.9	186	43.5	584	38.7	
	No neoadjuvant therapy in contradiction to guidelines	177	16.4	42	9.8	219	14.5	
Postoperative therapy	No adjuvant therapy according to guidelines	299	27.7	123	28.7	422	28.0	0.012
	Adjuvant therapy	437	40.5	204	47.7	641	42.5	
	No adjuvant therapy in contradiction to guidelines	318	29.5	95	22.2	413	27.4	
	No adjuvant therapy due to perioperative death	25	2.3	6	1.4	31	2.1	

Upper third = 12–16 cm, middle third = 6–11.9 cm, lower third = 0–5.9 cm from anal verge

**Fig. 1:**
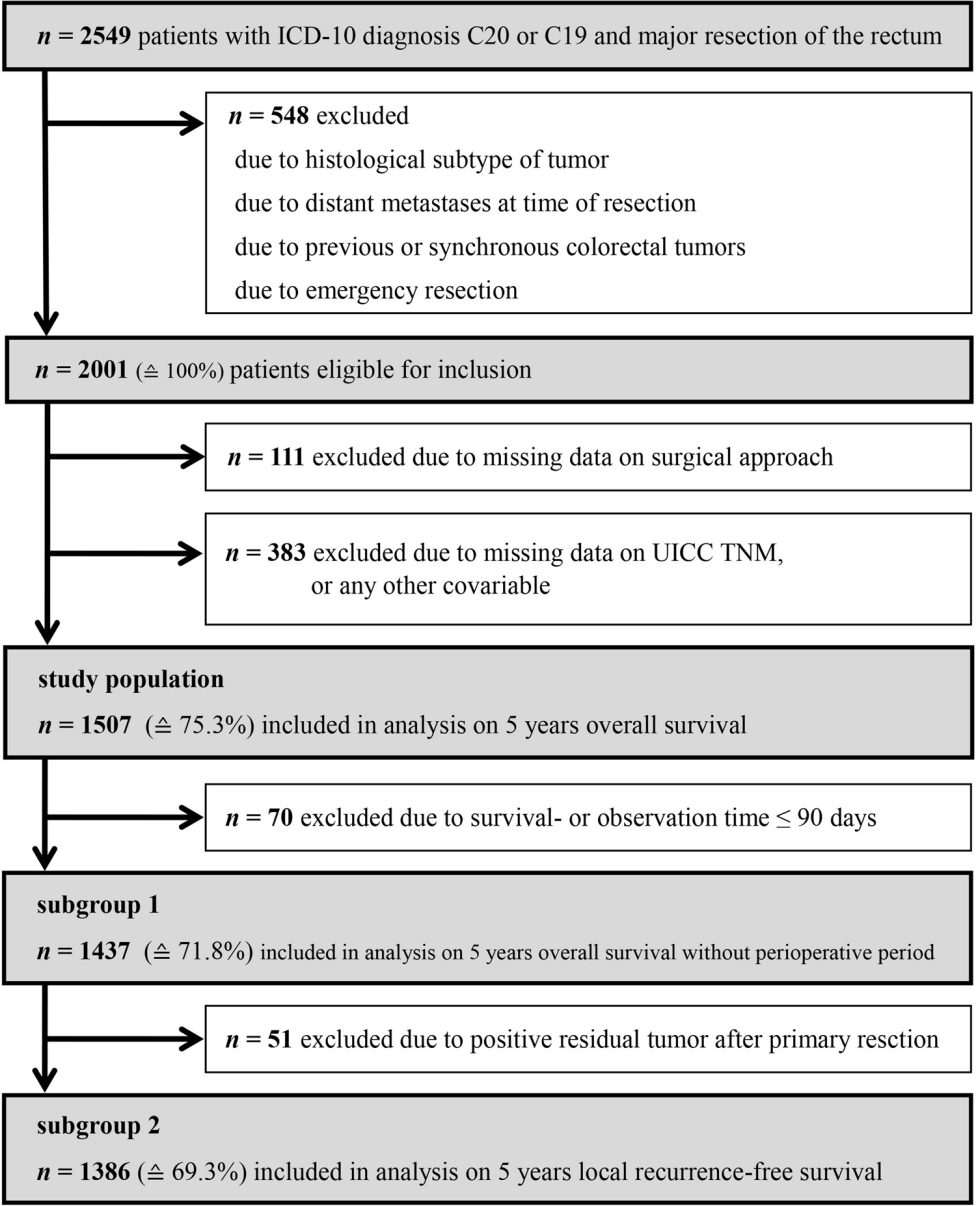
Flowchart of study patient selection

All analyses were conducted on an intention-to-treat basis, which means that conversions remain part of the laparoscopic group. The median follow-up time was 7.1 years. In order to compare survival rates in patients with laparoscopic versus open resections, a 5-year observation period after surgery was used. To focus on long-term oncologic outcomes, unless otherwise indicated, a 90-day cut-off time was applied to eliminate the effect of perioperative mortality. Patients with a survival or observation time of less than 91 days were excluded from these analyses (subgroup 1, Fig. 1). Accordingly, *t* = 91 days after surgery was set as the starting point for the observation time. For all analyses dealing with local recurrence-free survival, patients with positive resection margins were additionally excluded (subgroup 2, Fig. 1), as it would not have been possible to tell whether a real recurrence event had occurred or not in these patients. In addition to Kaplan–Meier analysis, multivariate Cox regression models [10] were used. All variables with a probability of less than *p* = 0.5 in χ^2^ tests (separately conducted for subgroups 1 and 2) of being equally distributed in the open and the laparoscopic surgery groups are regarded as potential confounders which must be adjusted for. The numbers of removed lymph nodes and R classification are deliberately not part of any multivariate model, since these can be regarded as surrogate parameters for the quality of a surgical procedure. To adjust for regional life expectancy as well as age- and gender-distribution, a relative survival model was computed. The underlying data on general mortality of the German population originate from the Human Mortality Database of the Max Planck Institutes [11]. To account for bias due to missing data, a sensitivity analysis was performed with inclusion of initially excluded patients. All significance tests were two-sided with a significance level of 0.05; results are displayed as *p* values or 95% confidence intervals (CI). The findings of this survey are presented in strict compliance with the Strengthening the Reporting of Observational studies in Epidemiology (STROBE) statement [12]. During this study, IBM SPSS 23 (IBM Corp., SPSS for Windows, Armonk, NY, USA), as well as R version 3.3.2 (R Foundation for Statistical Computing, Vienna, Austria; http://www.R-project.org/), and the R package “relsurv” (Maja Pohar-Perme [13]) were used.

## Results

Between January 1, 2004 and December 31, 2013, 2549 patients were diagnosed with a malignant tumor of the rectum or the rectosigmoid transition zone, and therefore received surgery. For the purpose of this study, 548 patients had to be excluded because they did not match the initially described criteria. Of the remaining patients eligible for inclusion, 111 could not be considered due to an unknown surgical approach. Another 383 patients had missing data on other important variables and were therefore also excluded from the analysis (Fig. 1).

**Fig. 2:**
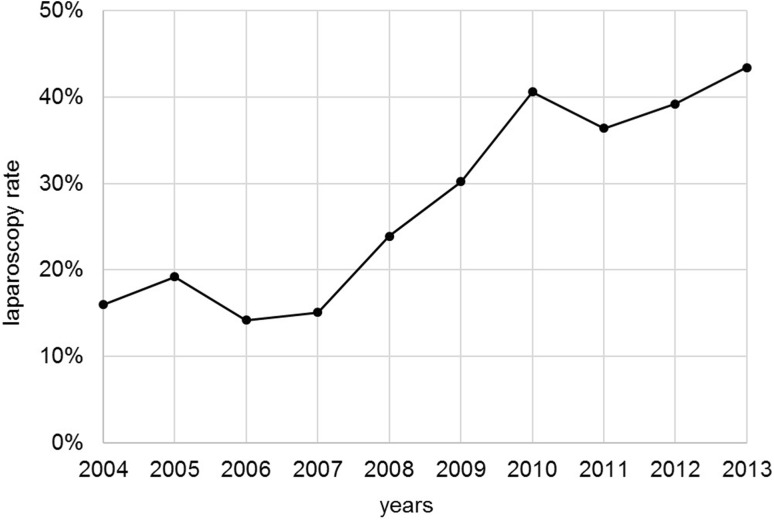
Laparoscopy rate

Among 1507 included patients, 28.4% underwent laparoscopic procedures, with the rate increasing over time from 16.0% in 2004 to 43.4% in 2013 (Fig. 2). Compared to the open resection group, laparoscopy patients were younger by 2.9 years on average, with age groups significantly differently distributed among the comparison groups (*p* < 0.001). Laparoscopic procedures were more likely to be performed in the upper or middle rectum than in the lower third (*p* < 0.001). Additionally, it is highly evident that the proportion of resections conducted in specialized colorectal cancer centers [14, 15] is higher in the laparoscopic than in the open surgery group (92.3 vs. 84.3%, *p* < 0.001). Laparoscopically treated patients are also more likely to receive pre- (*p* = 0.010) or postoperative (*p* = 0.012) chemoradiotherapy in accordance with guidelines. However, according to *χ*^2^ tests, there were no significant differences in terms of the distribution of grading (*p* = 0.114) or TNM stage (*p* = 0.256; Table 1).

**Fig. 3:**
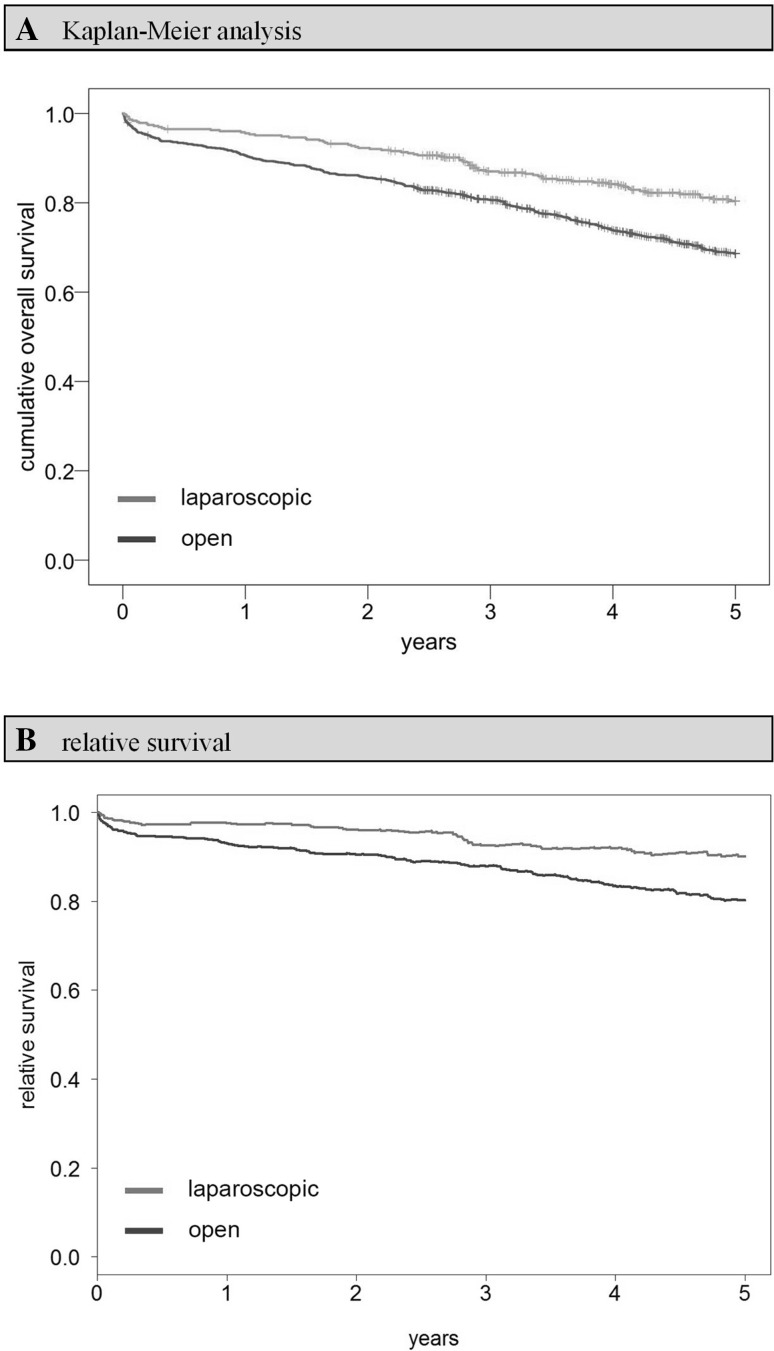
Overall survival including perioperative period (0 days–5 years). **A** Kaplan–Meier analysis: 5-year cumulative overall survival rate open versus laparoscopic: 68.6 versus 80.4%, *p* < 0.001. **B** relative survival analysis: 5-year relative survival rate open versus laparoscopic: 80.3 versus 90.2%, *p* < 0.001

**Fig. 4:**
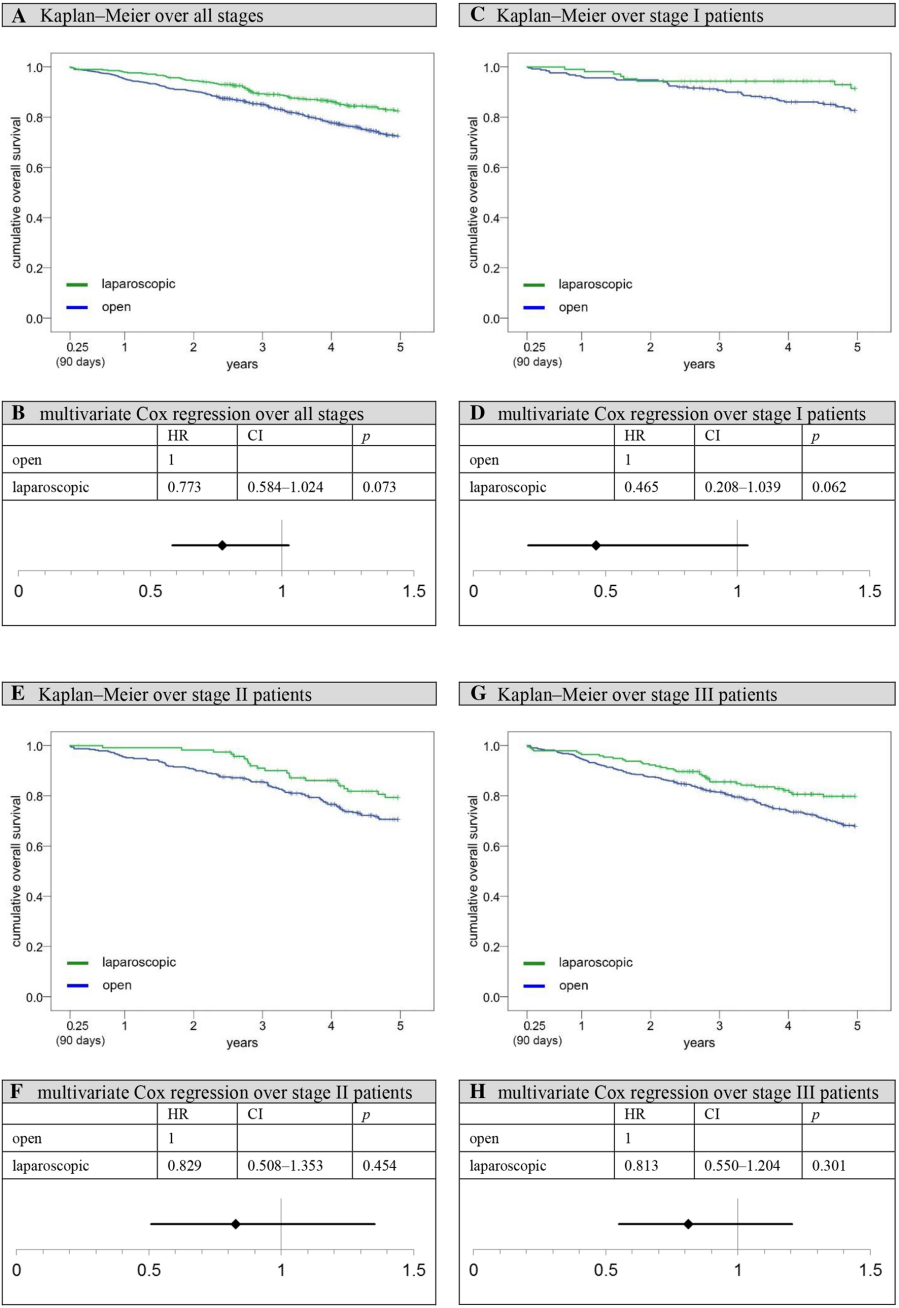
Overall survival after perioperative period (91 days–5 years). *HR* hazard ratio, *CI* two-sided 95% confidence interval. **A** Kaplan–Meier analysis over all stages: 5-year cumulative overall survival rate open versus laparoscopic: 72.5 versus 82.5%, *p* < 0.001. **B** Multivariate Cox regression analysis over all stages, adjustment for age, previous carcinomas, synchronous carcinomas, location, grading, TNM stage, hospital classification, resection group, and pre- and postoperative therapy; reference: open approach. **C** Kaplan–Meier analysis over stage I patients: 5-year cumulative overall survival rate open versus laparoscopic: 82.7 versus 91.4%, *p* = 0.047. **D** Multivariate Cox regression analysis over stage I patients, adjustment for age, previous carcinomas, synchronous carcinomas, location, grading, hospital classification, resection group, and pre- and postoperative therapy; reference: open approach. **E** Kaplan–Meier analysis over stage II patients: 5-year cumulative overall survival rate open versus laparoscopic: 70.6 versus 79.3%, *p* = 0.052. **F** Multivariate Cox regression analysis over stage II patients, adjustment for age, previous carcinomas, synchronous carcinomas, location, grading, hospital classification, resection group, and pre- and postoperative therapy; reference: open approach. **G** Kaplan–Meier analysis over stage III patients: 5-year cumulative overall survival rate open versus laparoscopic: 67.9 versus 79.8%, *p* = 0.010. **H** Multivariate Cox regression analysis over stage III patients, adjustment for age, previous carcinomas, synchronous carcinomas, location, grading, hospital classification, resection group, and pre- and postoperative therapy; reference: open approach

Comparing the Kaplan–Meier overall survival rates of open and laparoscopically treated patients up until 5 years after surgery, a benefit can be observed for the latter group (open vs. laparoscopic: 68.6 vs. 80.4%, *p* < 0.001; Fig. 3). The situation is similar when comparing 5-year relative survival rates (open vs. laparoscopic: 80.3 vs. 90.2%, *p* < 0.001; Fig. 3). These results remain stable after defining *t* = 91 days after surgery as the new starting point, while simultaneously excluding all patients who died perioperatively or whose observation time was shorter than 91 days (subgroup 1, Fig. 1). In this setting, the 5-year overall survival rate is 72.5% for open surgery and 82.5% for laparoscopy patients (*p* < 0.001), with significant advantages for laparoscopy particularly in Union for International Cancer Control (UICC) TNM stages I (5-year overall survival rate, open vs. laparoscopic: 82.7 vs. 91.4%, *p* = 0.047) and III (5-year overall survival rate, open vs. laparoscopic: 67.9 vs. 79.8%, *p* = 0.010) if analyzed separately (Fig. 4). With UICC TNM stage II patients, the survival benefit did not reach significance (5-year overall survival rate, open vs. laparoscopic: 70.6 vs. 79.3%, *p* = 0.052). Moreover, a multivariate Cox regression analysis was conducted. Applying the previously described methodology to account for all potentially unequally distributed confounders, it was adjusted for age, previous carcinomas, synchronous carcinomas, location, grading, TNM stage, hospital classification, resection group, preoperative treatment, and postoperative therapy. Having done so, a survival benefit for laparoscopically treated patients remained, although the significance level was narrowly missed (hazard ratio, HR 0.773; 95% CI 0.584–1.024; *p* = 0.073; Fig. 4). Contemplating HRs for each TNM stage separately, stage I patients seemed to benefit most from laparoscopy (HR 0.465, 95% CI 0.208–1.039; *p* = 0.062; Fig. 4). In the course of sensitivity analysis, it became evident that the necessary exclusion of patients with missing data obviously did not favor the laparoscopic group, since excluded open surgery patients had worse survival rates than their laparoscopic counterparts. The difference, however, was not significant (5-year overall survival rate open-excluded vs. laparoscopic-excluded: 72.9 vs. 76.5%, *p* = 0.529).

**Fig. 5:**
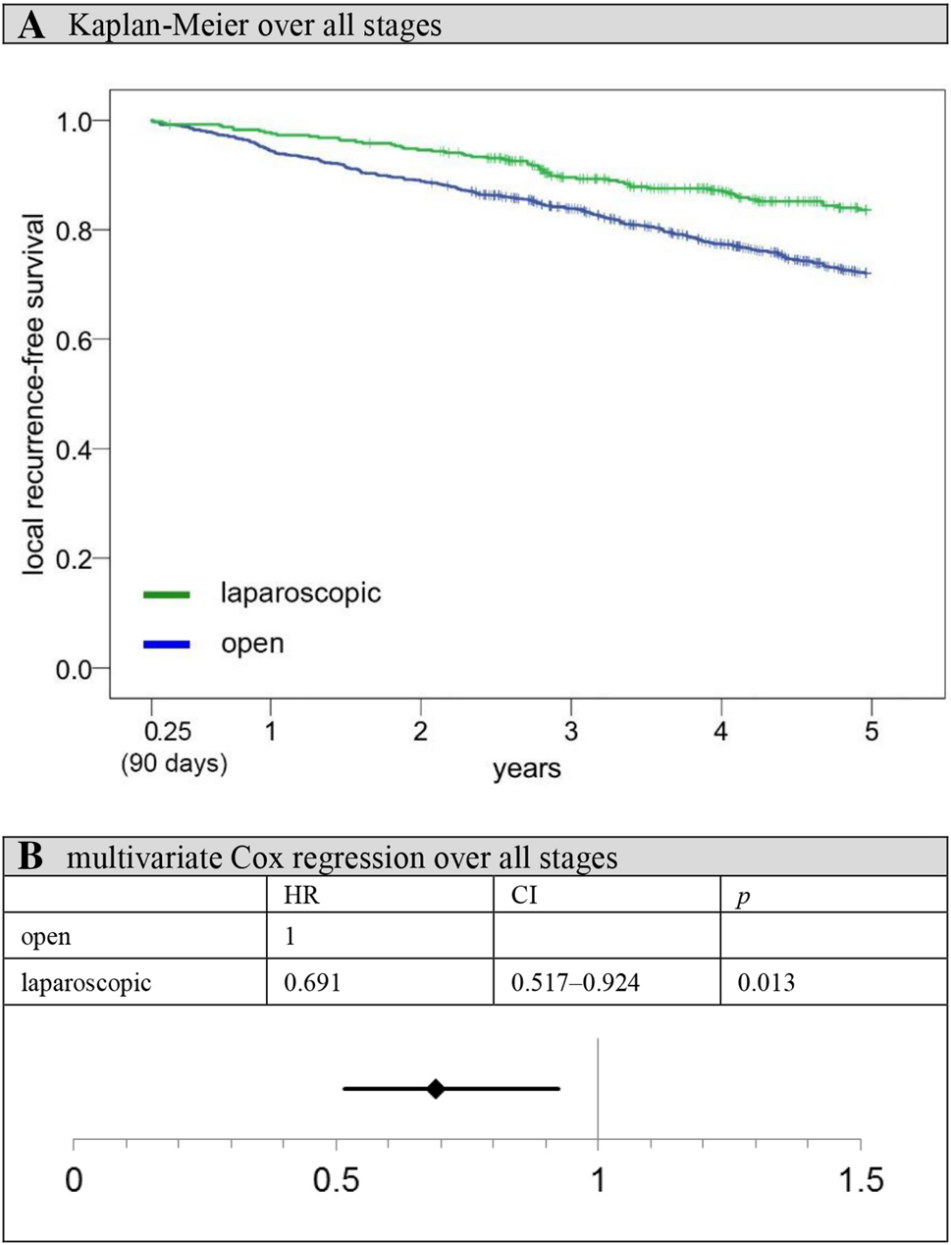
Local recurrence-free survival (91 days–5 years). *HR* hazard ratio, *CI* two-sided 95% confidence interval. **A** Kaplan–Meier analysis over all stages: 5-year recurrence-free survival rate open versus laparoscopic: 72.1 versus 83.6%, *p* < 0.001. **B** Multivariate Cox regression analysis over all stages, adjustment for age, synchronous carcinomas, location, grading, TNM stage, hospital classification, resection group, and pre- and postoperative therapy; reference: open approach

When evaluating disease-free survival rates, comparable results are observed. The following analyses were all restricted to an observation time starting at *t* = 91 days and patients with an initially complete tumor resection (subgroup 2, Fig. 1). Again, there is a significant advantage for laparoscopically operated patients in Kaplan–Meier analysis (5-year disease-free survival rate open vs. laparoscopic: 68.1 vs. 77.3%, *p* = 0.003). After adjustment for age, previous carcinomas, synchronous carcinomas, location, grading, TNM stage, hospital classification, resection group, preoperative treatment, and postoperative therapy, the benefit for laparoscopically treated patients no longer reached the significance level (HR 0.842, 95% CI 0.657–1.078, *p* = 0.173). The situation changes slightly when concentrating on local recurrence-free survival (5-year local recurrence-free survival rate open vs. laparoscopic: 72.1 vs. 83.6%, *p* < 0.001; Fig. 5). This time the observed benefit of laparoscopy remained stable even after adjusting for age, synchronous carcinomas, location, grading, TNM stage, hospital classification, resection group, preoperative treatment, and postoperative therapy (HR 0.691, 95% CI 0.517–0.924; *p* = 0.013; Fig. 5). Sensitivity analysis shows that excluded open patients have worse survival rates than excluded laparoscopy patients (5-year local recurrence-free survival rate open-excluded vs. laparoscopic-excluded: 70.9 vs. 74.7%, *p* = 0.527). Therefore, the superior laparoscopic group was once again not favored by the exclusion process.

## Discussion

The comparison of laparoscopic and open surgery for rectal cancer has been subjected to clinical research for many years now. A variety of studies focusing on perioperative endpoints such as length of hospital stay, mortality within 30 days, or lymph node removal already exist. According to these publications, laparoscopy, for the most part, is followed by favorable or at least equivalent results [16–19]. Although an increasing number of rectal carcinoma resections are performed laparoscopically, concerns persist regarding the equality of oncological resection in comparison to the open approach. This discussion was initiated by the outcomes of the CLASICC trial, which indicated a non-significantly higher rate of circumferential resection margin positivity in patients with laparoscopic anterior resection (open vs. laparoscopic: 6.3 vs. 12.4%, difference 6.1% points, 95% CI − 2.1 to 14.4). In contrast to these findings, the long-term outcomes of the CLASICC trial showed no difference in overall survival and locoregional recurrence rate when comparing laparoscopic with open rectal cancer surgery after 3 and 5 years. In 2015, the COLOR II study group published their randomized trial’s findings on 3-year overall and disease-free survival [20]: according to Kaplan–Meier analysis, the minimally invasive approach is associated with superior overall survival rates (difference 3.1% points, 95% CI 1.6–7.8). Locoregional recurrence rates are 5.0% in both groups. However, it would be problematic to simply transfer these results to daily clinical practice. RCTs are often conducted in ambitious clinics providing ideal conditions and showing a strong interest in new techniques. To evaluate whether laparoscopy is a safe alternative for rectal cancer surgery in an everyday setting, large population-based surveys like this one are required. Since results on 5-year survival in the COLOR II trial collective are still pending, a direct comparison of the studies is not possible. However, matching Kaplan–Meier overall survival rates at *t* = 3 years, similar advantages for laparoscopy are seen in both surveys. In the COLOR II trial, superiority of laparoscopy was observed particularly in stage III patients, while in this study, advantages were observed in all stages. It should, however, be borne in mind that analyzing simple Kaplan–Meier survival rates of observational studies can never be accurate enough.

The price that retrospective surveys have to pay in exchange for depicting reality is a variable amount of indication bias. Patients tend to receive one or the other treatment because of certain reasons. Based on the US National Cancer Database, Nussbaum et al. could, for example, show that younger age—as in the present study—is a significant predictor of the laparoscopic approach (odds ratio, OR 0.94; *p* < 0.001), while larger tumors and higher tumor stages are found more frequently among open resections [21]. Furthermore, patients with tumors of the upper or middle rectum and patients of specialized colorectal cancer centers were more likely to undergo laparoscopy in this study. Stratification by stage or other important variables is a useful strategy to correct for different risk profiles between groups. In order to be able to consider various inhomogeneities simultaneously, multivariate Cox regression analysis was applied. Thus, it could be adjusted for variables such as age, tumor stage, or additional therapies. Unfortunately, there was no information available on non-oncologic comorbidities, which is an important limitation of this survey. Patients with severe comorbidities have an increased mortality risk independently of their neoplastic disease [22, 23]. However, several studies confirmed an association between age and the number of a person’s comorbidities [24, 25]. Therefore, adjustment for age can contribute to balance the inequality between the laparoscopic and the open groups. Nevertheless, thorough information about all of patient’s comorbidities would facilitate an even more accurate risk adjustment. In this context, missing data is another important issue. If it was not possible to fill information gaps, patients had to be excluded to match mandatory statistical standards. However, sensitivity analysis could show that the necessary exclusion process did not favor the superior group. Thus, all presented findings can be regarded as very stable.

The aim of this study was to paint a holistic picture of rectal cancer surgery. Therefore, only the most necessary inclusion criteria were applied. It was not the intention of this study to present sophisticated results only relevant for highly selected subgroups under certain circumstances. An intention-to-treat analysis was deliberately chosen. With less experienced surgeons—who also form part of daily clinical practice—conversions are not uncommon and have to be regarded as a regular consequence of laparoscopic surgery. Taking everything into account, it becomes obvious that population-based retrospective surveys like the present investigation cannot produce exact results; they are, however, indispensable for showing trends toward superiority or inferiority after daily implementation of new techniques.

## Conclusion

Within the population evaluated here, laparoscopic removal of rectal tumors proved to be a safe alternative to the conventional approach. This study demonstrates that survival after laparoscopic resection is at least equivalent to open surgery, even outside the setting of a randomized trial. To further confirm this insight, more similar studies based on comprehensive cancer registries are required.
